# Unlocking Better Keloid Treatment: Corticosteroid, 5‐Fluorouracil, and Hyaluronidase vs. Corticosteroid Alone – A Randomized Comparative Study

**DOI:** 10.1002/hsr2.70883

**Published:** 2025-05-26

**Authors:** Ala Ehsani, Fatemeh Lotfi, Alireza Firooz, Amirhoushang Ehsani, Zahra Razavi, Mahshid Sadat Ansari, Pedram Nourmohammadpour, Fatemeh Sima, Mina Koohian Mohammadabadi, Amirhossein Rahimnia

**Affiliations:** ^1^ Student Research Committee, Faculty of Medicine Shahid Beheshti University of Medical Sciences Tehran Iran; ^2^ School of Medicine Iran University of Medical Sciences Tehran Iran; ^3^ Center for Research & Training in Skin Diseases & Leprosy Tehran University of Medical Sciences Tehran Iran; ^4^ Department of Dermatology, Razi Hospital Tehran University of Medical Sciences Tehran Iran; ^5^ Autoimmune Bullous Diseases Research Center, Razi Hospital Tehran University of Medical Sciences Tehran Iran; ^6^ Students' Scientific Research Center (SSRC) Tehran University of Medical Sciences Tehran Iran

**Keywords:** 5‐fluorouracil, corticosteroids, hyaluronidase, hypertrophic scar, keloid

## Abstract

**Background and Aims:**

Keloids are challenging lesions to manage due to their resistance to treatment and high recurrence rates. This study evaluated the efficacy of an intralesional injection combining triamcinolone acetonide, 5‐fluorouracil, and hyaluronidase compared with triamcinolone monotherapy in treating keloids.

**Methods:**

This single‐blinded clinical trial involved 16 participants, block‐randomized into two groups. The intervention group received intralesional injections of triamcinolone acetonide (0.2 cc of 40 mg/cc solution), 5‐fluorouracil (0.4 cc of 250 mg/5cc vial), and hyaluronidase (0.2 cc equivalent to 300 IU). The control group received triamcinolone acetonide (0.2 cc of 40 mg/cc solution) with 0.6 cc lidocaine of 2%. Keloid characteristics were assessed before treatment, after three injections at 3–4‐week intervals, and 2 months posttreatment.

**Results:**

Significant improvements were observed in both groups. However, the intervention group showed greater reductions in lesion height, pliability, and modified Vancouver scar scale scores compared to the control group. No complications resulted from our interventional injection during the study period, while telangiectasia occurred with triamcinolone monotherapy.

**Conclusion:**

These findings suggest that triple therapy was as effective as steroid monotherapy, with better outcomes in specific aspects of scar improvement and without side effects. Hyaluronidase could be promising for further research in this area.

## 1 Introduction

Keloid, a benign fibroproliferative skin tumor, results from excessive growth of granulation tissue or collagen type three in the process of healing, following cutaneous injury, irritation, or even spontaneously [1, 2]. This lesion can overgrow from the border of injury and form an ill‐defined, bizarre plaque, which can first appear at any time after the irritation but is generally observed around 3 months post‐injury [3, 4]. Besides its psychological sequelae as a cosmetic concern, individuals may experience various degrees of pain and pruritus, affecting their quality of life, which highlights the importance of managing it [5].

The remarkable point about its management is the high rate of keloid recurrence, which makes it a challenging condition in the field of dermatology. To the best of our knowledge, there is no permanent, proven treatment that can resolve this lesion completely [6, 7]. However, there are various possible options for at least controlling it, such as surgery, radiation and pharmaceutical therapies like intralesional injection [3].

Intralesional injections, as a minimally invasive intervention, are the most considerable methods and the injection of corticosteroids, especially triamcinolone acetonide, is the key modality [3, 8]. Although this approach is still a highly effective way, cases have been reported with resistance to corticosteroid use [7, 9]. Also, beyond the pain during injection, up to 63% of patients could suffer from complications like skin atrophy, depigmentation, and telangiectasias [10]. Due to these side effects and the chance of recurrence in steroid use, an increased tendency to find another component has emerged.

Different drugs have been studied as substitutes for steroids, like 5‐fluorouracil, verapamil, and platelet‐rich plasma [8]. But still the role of corticosteroids in decreasing inflammation, suppressing collagen and glycosaminoglycan synthesis, reducing fibroblast development and enhancing collagen degradation is undeniable [11]. Therefore, the best approach would be the combination therapy that benefits from steroid advantages while minimizing the drawbacks by reducing the dosage.

Some studies have been conducted to assess the combination of steroid and 5‐fluorouracil as well as corticosteroids with verapamil and found their superiority to steroid monotherapy [12, 13]. However, still much more needs to be done to discover new approaches.

One of the promising agents that could be used in this context is hyaluronidase. It has been shown that hyaluronic acid induces IL‐1 production, which leads to fibroblast proliferation and an increase in collagen production [14]. Therefore, using hyaluronidase as a hyaluronic acid splitter could break this trend. Also, hyaluronidase has a spreading factor by increasing permeability, which could increase the effect of other drugs used in combination with it [15].

In this study, we evaluate the intralesional therapy combination of triamcinolone acetonide, 5‐fluorouracil and hyaluronidase compared to triamcinolone acetonide alone. By finding a more efficient injection method, not only will the patient's discomfort be minimized, but also the psychological consequences of the lesion as a cosmetic burden would be reduced. Also, more persistent management for such a condition with a high rate of recurrence would lead to the efficiency of health system.

## 2 Materials and Methods

This study was a single‐blinded clinical trial in which participants were block‐randomized into parallel groups to compare the efficacy of combination intralesional injection of corticosteroids, 5‐fluorouracil, and hyaluronidase versus only‐corticosteroids in patients with keloid.

The trial was conducted at Razi Hospital, a referral dermatology hospital in Tehran, Iran, between March 2021 and March 2022. In this period, 16 patients over the age of 18, with a diagnosis of keloid of at least 3 months' duration, who had not received intralesional treatment in the previous 3 months, were included. All participants provided informed consent and agreed to adhere to the study procedures and follow‐up requirements. Before enrollment, all conditions were explained to eligible patients, and they were free to withdraw from the study at any time. Exclusion criteria included pregnant or breastfeeding women, individuals with known allergies to corticosteroids, 5‐fluorouracil, or hyaluronidase, as well as those using immunosuppressive drugs or systemic corticosteroids within the last 3 months. Patients with active infections, uncontrolled chronic conditions such as diabetes or autoimmune diseases, and those with a history of radiation therapy to the affected area were also excluded. Additionally, individuals unwilling or unable to comply with the study's follow‐up schedule or procedures were not included.

Participants were block‐randomly assigned to two groups to conduct a structurally equivalent study. In the intervention group (A), patients received intralesional injections of 300 IU hyaluronidase (0.2 cc), 0.4 cc 5‐fluorouracil from 250 mg/5cc vial (20 mg), and 8 mg triamcinolone acetonide (0.2 cc from 40 mg/cc solution) through 27‐gauge needle via an insulin syringe. The injections were administered over three sessions, spaced 3–4 weeks apart (within a 3‐month period), by the same experienced dermatologist. All procedures in the control group (B) were the same, except the mono‐injection of triamcinolone acetonide into the lesion. To prepare a solution of TAC in the control group, 0.6 cc lidocaine 2% was added to 0.2 cc triamcinolone acetonide suspension with a concentration of 40 mg/cc. The final solution was 0.8 cc of liquid, containing 8 mg TAC, the same volume as the intervention group.

To evaluate the outcome without bias, photographs and scar status were recorded and assessed by a blinded dermatologist at three‐time points: before the intervention, at the end of the treatment period, and 2 months posttreatment. Scar‐related variables, including size, height, color, and pliability of the scars, as well as the presence of atrophy, telangiectasia, hypo/hyperpigmentation, and ulcer, were evaluated by the blinded dermatologist, while the state of pain, and pruritus were scored according to patients' statements. The modified Vancouver scare scale was calculated based on its own recognized criteria, which evaluated the lesion's vascularity, pigmentation, pliability, height, pain, and pruritus with an overall score of 0–16 [16]. A vernier caliper was used to measure the size and height of scars, and the obtained numbers were reported in mm^2^ and mm, respectively. The scoring system used for other variables, as well as MVSS, is summarized in Tables 1 and 2, respectively.

**Table 1:** Variables scoring system.

Dermatologist judgment	
Color	
Perfect	0
Slight mismatch	1
Obvious mismatch	2
Pliability	
Supple	0
Firm	1
Rope	2
Atrophy	
Absent	0
Present	1
Telangiectasias	
Absent	0
Present	1
Pigmentation	
Absent	0
Present	1
Ulcer	
Absent	0
Present	1
Patients' statement	
Pain	
Absent	0
Present	1
Pruritus	
Absent	0
Present	1

**Table 2:** Modified Vancouver Scar Scale.

Modified Vancouver Scar Scale	
Vascularity	
Normal	0
Pink	1
Red	2
Purple	3
Pigmentation	
Normal	0
Hypopigmentation	1
Mixed	2
Hyperpigmentation	3
Pliability	
Normal	0
Supple	1
Yielding	2
Firm	3
Ropes	4
Contracture	5
Height	
Flat	0
< 2 mm	1
2–5 mm	2
> 5 mm	3
Pain	
None	0
Occasional	1
Required medication	2
Pruritus	
None	0
Occasional	1
Required medication	2
Total score	16

Additionally, a checklist containing variables such as age, gender, location of the lesion, duration, and reason of its appearance and history of previous treatment was filled based on patients' history.

The primary outcome of this study was to compare the effectiveness of the triple therapy with corticosteroid monotherapy in reducing the size, height, color, pain, and pliability of keloid scars. Additionally, improvements in pruritus, the modified Vancouver scar scale (MVSS) score, and overall lesion characteristics were evaluated. The secondary outcome involved analyzing patient background variables, such as age, gender, lesion location, and previous treatments, to assess their potential influence on treatment efficacy. Furthermore, the presence of complications, including atrophy, telangiectasia, pigmentation changes, and ulceration, was monitored across both treatment groups.

While recruiting patient in accordance with declaration of Helsinki, the trial was registered on the Iranian Registry of Clinical Trials (IRCT), under registration number IRCT20220115053715N1 on January 28, 2022, and was approved by the Research Ethics Committee of Tehran University of Medical Sciences under approval ID IR.TUMS.MEDICINE. REC.1400.1166.

All data were analyzed with SPSS (IBM SPSS Statistics, Armonk, NY, USA), version 28, with a statistical significance level of less than 0.05. Qualitative data were described using frequency and percentage, while quantitative data were summarized using mean, median, standard deviation (SD), and interquartile range (IQR). For further analysis, considering the limited number of cases, non‐parametric tests were used. The quantitative variables of the two groups were compared with Mann–Whitney, and the Fisher's exact test and likelihood ratio test were used for qualitative variables. For comparison of time point changes in each group, the Friedman test was utilized (Time 1: before treatment, Time 2: after treatment, Time 3: follow‐up).

## 3 Results

In this study, all 16 eligible patients were tracked until the end of their follow‐ups. Regarding background variables, there was no statistically significant difference between age, gender, location, duration, and reason of lesion, as well as previous treatment between patients of the two groups. Therefore, the distribution of cases was homogenous without disturbing factors (Table 3).

**Table 3:** Descriptive statistics of qualitative, quantitative variables.

Quantitative variables		Group		*p* value
		hyaluronidase, Steroid, 5‐fluorouracil	Steroid	
Gender	Male	5 (62.5)	4 (50)	P^a^ = 0.61
	Female	3 (37.5)	4 (50)	
Location of the keloid	Upper limb	2 (25)	0	P^b^ = 0.36
	Trunk	3 (37.5)	4 (50)	
	Chest	2 (25)	2 (25)	
	Face	1 (12.5)	1 (12.5)	
	Neck	0	1 (12.5)	
The cause of the lesion	Tattoo scar	1 (12.5)	1 (12.5)	P^b^ = 0.48
	surgical scar	4 (50)	4 (50)	
	Acne	2 (25)	1 (12.5)	
	Laser	1 (12.5)	0	
	Hidradenitis suppurativa	0	1 (12.5)	
	Spontaneously	0	1 (12.5)	
Previous treatment history	Yes	3 (37.5)	4 (50)	P^b^ = 0.61
	No	5 (62.5)	4 (50)	
Previous treatment	No	5 (62.5)	4 (50)	P^b^ = 0.61
	Topical steroid	2 (25)	3 (37.5)	
	Adalimumab	0	1 (12.5)	
	Topical steroid + Pulsed Dye Laser	1 (12.5)	0	
Qualitative variable		Mean (SD) Median (IQR) Range	Mean (SD) Median (IQR) Range	*p* value
Age (years)		37.63±9.66 46±10 25‐51	37.75±15.83 42.5±22 19‐65	P^c^ = 0.87
Duration (months)		10.5±10.87 38±18 3‐36	9.75±6.36 6±8 6‐24	P^c^ = 0.64

^a^ Exact Fisher test.

^b^ Likelihood Ratio test.

^c^ Mann–Whitney test.

To compare the effectiveness of these two methods with each other, the lesion's features were assessed before treatment, after treatment, and 2‐month post‐intervention.

At baseline, there was no statistically significant difference in size, height, pain, the pliability of the lesion and presence of pruritus, atrophy, telangiectasia, pigmentation, ulcer, as well as modified Vancouver scar scale score in two groups. The only variable with a significant difference at the initiation was the color of the keloid scar (*p *= 0.046).

Posttreatment analysis revealed that injection of steroid, Hyaluronidase, and 5‐fluorouracil compared to Steroid‐only was more successful in reduction of height and pliability, also in improvement of modified Vancouver scar score (*p* < 0.05). Although the pain score and pruritus in the new approach were reduced more in number, no statistical difference was found (*p* > 0.05).

All intergroup comparisons of 2‐month follow‐ups were the same as the posttreatment results.

Besides the comparison of Groups A and B, the effect of treatment was evaluated in each group separately. A significant difference was detected in reduction of size, height, color, pain, pliability, pruritus, and MVSS score comparing before and after treatment (comparing starting time to posttreatment as well as 2‐month follow‐up) in both groups.

At the beginning of the study, there was no evidence of atrophy, hypo/hyperpigmentation, ulcer, or telangiectasia in any of the participants. After the injections, only the emergence of telangiectasia was noticed in the group receiving steroid monotherapy (*p*‐value of 0.018 comparing this group before and after treatment).

Moving to comparing follow‐up time to posttreatment, no statistically significance difference in any of our parameters were found. All data is summarized in Table 4.

**Table 4:** Descriptive statistics of clinical in the study in each group.

Variable	Group	Mean (SD) median IQR		
		Time 1	Time 2	Time 3
Size	Hyaluronidase, Steroid, 5‐FU	121 (22.84)	49.93 (9.36)	49.93 (9.36)
		30	7.5	7.5
		102.5	65.3	65.3
	Steroid	43.5 (34.63)	22.76 (19.8)	22.76 (19.8)
		45	17.5	17.5
		61.3	40.6	40.6
Height	Hyaluronidase, Steroid, 5‐FU	4.5 (2.39)	1.62 (0.96)	1.62 (0.96)
		3.5	1.26	1.26
		2	1.83	1.83
	Steroid	4 (1.69)	2.78 (1.37)	2.78 (1.37)
		3.5	2.31	2.31
		1	1	1
Color	Hyaluronidase, Steroid, 5‐FU	0.92 (0.10)	0.33 (0.05)	0.33 (0.05)
		1	0.30	0.30
		0.2	0.10	0.10
	Steroid	0.82 (0.7)	0.57 (0.13)	0.57 (0.13)
		0.8	0.56	0.56
		0	0.24	0.24
Pain	Hyaluronidase, Steroid, 5‐FU	0.58 (0.49)	0.17 (0.14)	0.17 (0.14)
		0.85	0.25	0.25
		1	0.30	0.30
	Steroid	0.38 (0.42)	0.22 (0.24)	0.22 (0.24)
		0.3	0.20	0.20
		0.8	0.44	0.44
Pliability	Hyaluronidase, Steroid, 5‐FU	0.95 (0.05)	0.35 (0.11)	0.35 (0.11)
		0.95	0.36	0.36
		0.10	0.06	0.06
	Steroid	0.91 (0.03)	0.64 (0.12)	0.64 (0.12)
		0.90	0.67	0.67
		0	0.19	0.19
Pruritus	Hyaluronidase, Steroid, 5‐FU	0.75 (0.46)	0.23 (0.20)	0.23 (0.20)
		1	0.25	0.25
		1	0.35	0.35
	Steroid	0.50 (0.53)	0.28 (0.32)	0.28 (0.32)
		0.50	0.25	0.25
		1	0.50	0.50
Atrophy	Hyaluronidase, Steroid, 5‐FU	0	0	0
	Steroid	0	0	0
Telangiectasia	Hyaluronidase, Steroid, 5‐FU	0	0	0
	Steroid	0	0.5 (0.53)	0.5 (0.53)
		0	0.5	0.5
		0	1	1
Hypo/hyperpigmentation	Hyaluronidase, Steroid, 5‐FU	0	0	0
	Steroid	0	0	0
Ulcer	Hyaluronidase, Steroid, 5‐FU	0	0	0
	Steroid	0	0	0
Modified Vancouver scar scale	Hyaluronidase, Steroid, 5‐FU	12 (2)	4.88 (1.35)	4.88 (1.35)
		12.5	5	5
		4	2	2
	Steroid	10.25 (2.12)	7.13 (1.64)	7.13 (1.64)
		10.5	7.5	7.5
		5	4	4

## 4 Discussion

As a refractory lesion, the treatment of keloid is not straightforward and the recurrence is inevitable.

To find a novel approach, we evaluated the effectiveness of combination therapy with steroid, 5‐fluorouracil, and hyaluronidase compared to steroid‐only injection. The new method resulted in a significant reduction in the size and height of the lesion, improvement of its color and pliability, suppression of pain, pruritus, and modified Vancouver scar scale score like the traditional method using steroid alone. No side effect has been found in the former, but the emergence of telangiectasia has been seen in the latter approach of our study.

When comparing the two methods, the reduction of height of the scar, improvement of calculated modified Vancouver scar scale and softening of the lesion were statistically significance. Although, a statistical difference has been found in matter of color too, because of the presence of this variety at the starting point, it could not be contributed to our intervention for sure.

It was shown in previous studies that the injection of 5‐fluorouracil led to improvement of pain and pruritus [5, 17]. Despite the alleviating effect of our method on these symptoms, the reduction was not statistically significant among our two groups, which could be due to the limited number of cases studied. Furthermore, these two variables are subjective, and the tolerance threshold of them varies from person to person. As our participants were not blinded, these findings could be the result of bias following the knowledge of patients that they were receiving a new therapy.

Previous research on the efficacy of 5‐fluorouracil intralesional injection has revealed its role in softening and flattening of the scar. In a study conducted by Fitzpatrick, the author's 9‐year experience showed the significant role of 5‐FU in the inhibition of fibroblast proliferation, which led to its positive outcomes [18]. Also, a systematic review and meta‐analysis done by Jiang et al. showed that the combination of triamcinolone acetonide (TAC) and 5‐FU is superior to the injection of TAC alone [13]. This knowledge is consistent with our study in the reduction of height and improvement of softness in the group containing 5‐FU.

In our research, there was no statistically significant difference in matter of atrophy, hyper/hypopigmentation and ulcer formation between two groups while these adverse effects of steroids have proven [10]. This could be due to limited sessions of injections as well as short‐time follow‐up. On the other hand, in our conducted study, TAC mono‐injection caused telangiectasia while the combination method did not lead to this complication.

So, both steroid and 5‐FU have proven side effects. Steroids could cause skin atrophy, depigmentation and telangiectasias, and injection of 5‐FU is associated with pain and the risk of ulcer formation [10, 18]. Therefore, adding another effective component could be useful. Not only could we benefit from the advantages, but it would also reduce the required dose of other substances which means lower risk of side effects emergence.

In theory, hyaluronidase's role in the reduction of IL‐1 formation and, as a result, fibroblast and collagen synthesis inhibition make it a potential agent that could possibly stop overgrowth and keloid formation [14]. According to our data, the information about the effectiveness of hyaluronidase injection is too limited. In a study carried out by Nilesh et al., the triple combination of steroid, 5‐FU and hyaluronidase was studied, especially its role in softening the lesion and making other injections easier for clinicians was highlighted [19], which was consistent with our findings.

The intervention group of our study has received combination of 5‐FU and Hyaluronidase added to triamcinolone acetonide that make difficult to determine which agent caused the differences. Therefore, carrying out research in which only addition of hyaluronidase is assessed would make it easier to make conclusion.

Also, by determining longer follow‐up times, the performance of this new substance in rate of recurrence and occurrence of side effects can be interpreted more clearly. The other suggestion, is planning of double or even triple‐blinded clinical trials, as a result the risk of bias would be minimized as much as possible.

Although this study was limited in the number of participants, it showed that conducting a multi‐arm randomized clinical trial with a larger sample size evaluating the efficacy of hyaluronidase compared to other agents would be worthwhile to make a solid conclusion and introduce this agent as a promising method to overcome these resistant and hard‐to‐treat scars.

## 5 Conclusion

In conclusion, the primary goal of this study was to evaluate the combined injection of steroid, 5‐FU, and hyaluronidase into the keloid lesion compared to steroid‐alone. The findings demonstrated the efficacy of this new approach as well as its dominance over mono injection of triamcinolone acetonide in reducing height, pliability, and MVSS of keloids in our study. Furthermore, no complications resulted from our interventional injection. Considering hurdles of keloid management, this method could potentially bring significant advancements in this field.

## Author Contributions

**Ala Ehsani:** data curation, formal analysis, investigation. **Fatemeh Lotfi:** data curation, formal analysis, investigation, writing – original draft, writing – review and editing. **Alireza Firooz:** conceptualization, project administration, supervision, writing – review and editing. **Amirhoushang Ehsani:** conceptualization, project administration, supervision, writing – review and editing. **Zahra Razavi:** formal analysis, methodology, supervision, validation, writing – original draft. **Mahshid Sadat Ansari:** formal analysis, methodology, supervision, validation, writing – review and editing. **Pedram Nourmohammadpour:** conceptualization, supervision, writing – review and editing. **Fatemeh Sima:** data curation, formal analysis, methodology. **Mina Koohian Mohammadabadi:** data curation, methodology, writing – original draft. **Amirhossein Rahimnia:** conceptualization, investigation, methodology, project administration, writing – original draft, writing – review and editing.

## Conflicts of Interest

The authors declare no conflicts of interest.

## Transparency Statement

The lead author Amirhossein Rahimnia affirms that this manuscript is an honest, accurate, and transparent account of the study being reported; that no important aspects of the study have been omitted; and that any discrepancies from the study as planned (and, if relevant, registered) have been explained.

## Data Availability

The data that support the findings of this study are available from the corresponding author upon reasonable request.
